# Theissenolactone C Exhibited Ocular Protection of Endotoxin-Induced Uveitis by Attenuating Ocular Inflammatory Responses and Glial Activation

**DOI:** 10.3389/fphar.2018.00326

**Published:** 2018-04-09

**Authors:** Fan-Li Lin, Jau-Der Ho, Yu-Wen Cheng, George C. Y. Chiou, Jing-Lun Yen, Hung-Ming Chang, Tzong-Huei Lee, George Hsiao

**Affiliations:** ^1^Graduate Institute of Medical Sciences and Department of Pharmacology, School of Medicine, College of Medicine, Taipei Medical University, Taipei, Taiwan; ^2^Department of Ophthalmology, Taipei Medical University Hospital, Taipei, Taiwan; ^3^School of Pharmacy, College of Pharmacy, Taipei Medical University, Taipei, Taiwan; ^4^Department of Neuroscience and Experimental Therapeutics and Institute of Ocular Pharmacology, College of Medicine, Texas A&M Health Science Center, College Station, TX, United States; ^5^Department of Anatomy, School of Medicine, College of Medicine, Taipei Medical University, Taipei, Taiwan; ^6^Institute of Fisheries Science, National Taiwan University, Taipei, Taiwan; ^7^Ph.D. Program for the Clinical Drug Discovery from Botanical Herbs, College of Pharmacy, Taipei Medical University, Taipei, Taiwan

**Keywords:** endotoxin-induced uveitis, ocular inflammation, microglia, NF-κB, TNF-α

## Abstract

The aim of this study was to investigate the effects of a natural component, theissenolactone C (LC53), on the ocular inflammation of experimental endotoxin-induced uveitis (EIU) and its related mechanisms in microglia. Evaluation of the severity of anterior uveitis indicated that LC53 treatment significantly decreased iridal hyperemia and restored the clinical scores. Additionally, the deficient retina functions of electroretinography were improved by LC53. LC53 significantly reduced levels of tumor necrosis factor (TNF)-α, monocyte chemoattractant protein-1, protein leakage and activation of matrix metalloproteinases in the anterior section during EIU. Moreover, LC53 treatment decreased the oxidative stress as well as neuroinflammatory reactivities of GFAP and Iba-1 in the posterior section. Furthermore, LC53 decreased the phosphorylation of p65, expression of HSP90, Bax, and cleaved-caspase-3 in EIU. According to the microglia studies, LC53 significantly abrogated the productions of TNF-α, PGE_2_, NO and ROS, as well as inducible NO synthase and cyclooxygenase-2 expression in LPS-stimulated microglial BV2 cells. The microglial activation of IKKβ, p65 phosphorylation and nuclear phosphorylated p65 translocation were strongly attenuated by LC53. On the other hand, LC53 exhibited the inhibitory effects on JNK and ERK MAPKs activation. Our findings indicated that LC53 exerted the ocular-protective effect through its inhibition on neuroinflammation, glial activation, and apoptosis in EIU, suggesting a therapeutic potential with down-regulation of the NF-κB signaling for uveitis and retinal inflammatory diseases.

## Introduction

Uveitis, an ocular inflammation involving the uveal track, is the fifth most common cause of severe visual loss, and it accounts for up to 20% of legal blindness in the developed world (Chang and Wakefield, 2002; Durrani et al., 2004). The eventual visual impairment in uveitis patients has always been ascribed to the ocular tissue damage caused by amplification of the inflammatory processes because most uveitis is a chronic and relapsing inflammatory disorder (Yanai et al., 2014). However, the standard treatment with corticosteroid in uveitis requires further discussion because of its potential adverse effects, such as increased intraocular pressure, higher infection susceptibility, and increased risk of developing cataracts and glaucoma (Lee and Foster, 2010). Therefore, alternative treatments that are safer and longer lasting are needed.

**FIGURE 1:**
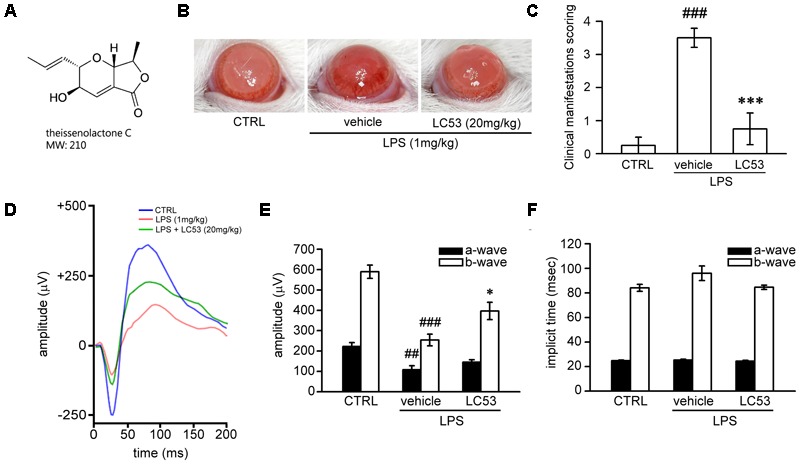
LC53 potentially protected against LPS-induced intraocular inflammation and prevented LPS-injured retinal function. **(A)** The chemical structure of LC53 (MW = 210). **(B)** Anterior ocular inflammation was evaluated by microscopic examination in anterior segment 72 h after footpad injection of lipopolysaccharide (LPS, 1 mg/kg). Representative ocular photography was taken at 72 h following LPS-induction in SD rat. **(C)** Clinical scoring for inflammation level of EIU in the absence (*n* = 4) or presence of LC53 (20 mg/kg, *n* = 4) and in control group (*n* = 4) were determined. **(D)** Representative scotopic ERG responses from control mice (blue curve), and EIU rat treated with the vehicle control (red curve) or LC53 (green curve) were recorded 1 day after LPS-injection. **(E,F)** Quantification of the average amplitudes and implicit time (time to peak) from control rat (*n* = 6), and EIU rat treated with the vehicle control (*n* = 6) or LC53 (*n* = 5). The ERG a-waves were negative, and the a-wave amplitudes were presented as the absolute value. The results were presented as the mean ± SEM. CTRL: control. ^##^*p* < 0.01, ^###^*p* < 0.001 compared with the control group treated with normal saline; ^∗^*p* < 0.05, ^∗∗∗^*p* < 0.001 compared with EIU group treated with vehicle.

Theissenolactone C, a fungal derivate extracted from *Theisseno cinerea* (Xylariaceae), was determined by its HR-ESI-MS and ^13^C-NMR data (MW = 210) (**Figure 1A**) (Liang et al., 2011). It has been reported that some species categorized in the genus *Xylaria* have been used in traditional Chinese medicine preparations, such as antimicrobial agents, antioxidant agents, and anti-inflammatory agents (Song et al., 2014), suggesting that derivatives of the Xylariaceae could probably be applied to drug development. It has been revealed that mixtures from *Theisseno cinerea* has significant growth-inhibitory activity against murine RAW264.7 macrophage cells (Liang et al., 2011). Our preliminary results also indicated that LC53 had an anti-inflammatory effect in the LPS-stimulated THP-1 monocytic cells. Herein, we investigated the protective efficacy of LC53 on an EIU rat model and its anti-ocular inflammatory mechanisms.

The model of EIU, which mimics the pathologies in human acute uveitis, has been widely used to clarify the mechanisms of innate inflammation and evaluate potential ocular anti-inflammatory agents (Rosenbaum et al., 1980). EIU can be induced by systemic injection of a sublethal dose of LPS, which causes bilateral acute ocular inflammation, such as elevated inflammatory cytokine production, inflammatory cell infiltration, heavy protein flare, and dilation of iris vessels in the anterior section of the eye. Moreover, retinal cell death, breakdown of the blood-ocular barrier, choroiditis, retinal vascular leukostasis, and inflammatory cell infiltration into the posterior section of the eyes were also found (Okumura et al., 1990). Disruption of the blood-retinal barrier could eventually cause cystoid macular oedema, the most important cause of visual impairment in uveitis (Rothova et al., 1996). The disruption of the BRB resulted in proliferation of Müller cells because Müller glial cells integrate the blood-retina barrier by linking the vessels and neurons (Shen et al., 2012). LPS-activated toll-like receptor (TLR)-4 signaling in cooperation with MyD88 could activate NF-κB and MAPKs, culminating in the synthesis and release of inflammatory mediators, such as cytokines, chemokines, adhesion molecules, ROS, and reactive nitrogen radicals during EIU (Li et al., 2010). Much evidence has shown that LPS activates NF-κB/TAK1 signaling in macrophages, which mediates both NF-κB and MAPK activation, and subsequent production of proinflammatory mediators (Irie et al., 2000; Dong et al., 2006). It was also found TAK1-binding proteins (TABs) play a crucial role in regulation of TAK1 and NF-κB activation (Kenny and O’Neill, 2008). Therefore, the specific TAK1 inhibition was shown to inhibit LPS-induced innate immune response (Xi et al., 2016).

Increased tumor necrosis factor (TNF)-α and monocyte chemoattractant protein (MCP)-1 expression levels have been described in patients with acute uveitis or in a rat EIU model (Santos Lacomba et al., 2001; Tuaillon et al., 2002). Numerous studies have suggested that anti-TNF-α agents, such as infliximab, are a therapeutic option for the prevention and treatment of uveitis (Cordero-Coma and Sobrin, 2015). TNF-α is pleiotropic cytokine that is a major modifier of inflammatory and immune reactions (Sugita et al., 2007). During experimental uveitis, TNF-α was found to recruit leukocytes to the eye, enhance leukocyte adhesion to the vascular endothelium, activate macrophages and infiltrating T cells, and promote apoptosis of both resident cells and infiltrating cells (Dick et al., 2004). Treatment with TNF-α inhibitor significantly decreased apoptosis in the vascular endothelium, ganglion cell, and inner nuclear layers as well as inactivated caspase-8 and -3 in EIU rats (Koizumi et al., 2003). On the other hand, up to 50% of patients with acute anterior uveitis (AAU) are HLA-B27 positive, and Wegscheider et al. (2005) reported that carriers of a CCL2 allele, coding for MCP-1, were significantly more likely in patients with HLA-B27 associated AAU than positive controls. MCP-1 has been shown to have chemoattractant properties for natural killer (NK) cells, mast cells, basophils, monocytes, and memory T cells. From the transgenic mice model, the severity of EIU was strongly reduced in MCP-1^-/-^ mice compared with the wild-type control group, and macrophages were no longer recruited into the eye (Tuaillon et al., 2002). Furthermore, MMP secretion and activation were found to be induced and modulated by TNF-α and interleukin (IL)-1α during uveitis. In the clinical setting, elevated levels of MMPs were detected in the aqueous humor of patients and animals with uveal inflammation (Di Girolamo et al., 1996). Accordingly, selective blocking of MMP-2 and MMP-9 significantly reduced the severity of experimental autoimmune uveitis (El-Shabrawi et al., 2004).

Oxidative stress has also been implicated in the pathogenesis of several ocular diseases, including uveitis (Kruk et al., 2015). Increased ROS could activate the redox-sensitive transcription factors such as NF-κB and AP-1. As a consequence, over-expression of inflammatory mediators, such iNOS and COX-2, which secrete NO and PGE_2_, respectively, exacerbates the oxidative stress and leads to dysregulated inflammation (Gloire and Piette, 2009). Furthermore, ROS regulate various genes that are involved in cytotoxicity. Therefore, reducing the oxidative stress may ameliorate tissue damage during inflammatory reactions (Yadav et al., 2011).

We hypothesized that LC53 may resolve the severity of EIU based on its anti-inflammatory effects. Therefore, we investigated the protective effect of LC53 in a rat model of EIU. Additionally, the effects of LC53 on the endotoxin-induced expression of inflammatory mediators and its anti-inflammatory mechanisms were investigated and clarified in a LPS-stimulated microglia cell line (BV-2).

## Materials and Methods

### Reagents and Antibodies

LC53 was provided by Professor Tzong-Huei Lee (National Taiwan University), and the purity was at least 95%, as evidenced from the NMR spectrum. LPS from *Escherichia coli* O127:B8 was purchased from Sigma-Aldrich (St. Louis, MO, United States). TLR4, p-p65, MyD88, β-actin, and mouse/rabbit IgG antibodies (Dylight 488) were from GeneTex (Irvine, CA, United States). Iba1 antibody was from Millipore (Temecula, CA, United States). GFAP antibody was from ProSci Inc. (Poway, CA, United States). HSP90 antibody and HIGHDEF^®^ IHC fluoromount were from Enzo Life Sciences (Farmingdale, NY, United States). Bcl2, Bax, caspase-3, p-TAK1, p-IKKα/β, p-ERK, ERK, p-JNK, and JNK antibodies were from Cell signaling (Beverly, MA, United States). TAB2 antibody was from Proteintech (Rosemont, IL, United States). IKKβ, iNOS, and p65 antibodies were from Santa Cruz (Dallas, TX, United States). COX-2 antibody was from Novus Biologicals (Littleton, CO, United States). Lamin B1 antibody was from Abcam (Cambridge, MA, United States). α-tubulin antibody was from Neomarkers (Fremont, CA, United States). Peroxidase-conjugated anti-rabbit/mouse secondary antibodies were from Jackson ImmunoResearch (West Grove, PA, United States). 4′, 6′-Diamidino-2-phenylindole (DAPI) was from AAT Bioquest, Inc. (Sunnyvale, CA, United States). 2′, 7′-Dichlorodihydrofluorescein diacetate (DCFH-DA) was from Cayman (Ann Arbor, MI, United States). TRIsure^TM^ reagent was from Bioline (London, United Kingdom). 2X One-tube RT-PCR mix was from Bioman (New Taipei City, Taiwan). Agarose was from GeneDirex (Las Vegas City, NV, United States). GelRed^TM^ nucleic acid gel stain was from Biotium (Fremont, CA, United States).

### Animals Care

Male Sprague-Dawley rat (250–270 g body weight) was obtained from BioLASCO Taiwan Co., Ltd. (Taipei, Taiwan). All animal experiments were conducted in strict agreement with the Association for Research in Vision and Ophthalmology (ARVO) statement for the use of animals in ophthalmic and vision research, and they were approved by the Institutional Animal Care and Use Committee of Taipei Medical University (Approval Number: LAC-2017-0182).

### Endotoxin-Induced Uveitis

According to the method of Qin et al. (2014), EIU was induced by subcutaneous injection of LPS with a dosage of 1 mg/kg into a single footpad. LPS from *Escherichia coli* O127:B8 was dissolved in sterile and pyrogen-free saline. For the LC53 treatment group, the rat was intraperitoneally injected with LC53 (20 mg/kg) dissolved in co-solvent (ethanol: cremophor: saline = 1: 1: 8) 5 min before LPS injection. For the controlled group, each rat was also intraperitoneally injected with co-solvent as mentioned.

### Ophthalmic Score of EIU

The intensity of anterior segment inflammation was examined at 72 h after LPS injection and following euthanization. Pictures of the anterior part that reflect the visualization were taken by a handle hold digital microscope. Ophthalmic scoring of the EIU was performed as previously described (Qin et al., 2014) with some modifications. The severity of the EIU was graded from 0 to 4 using the following scale: 0 = no inflammatory reaction; 1 = discrete inflammation of the iris and conjunctival vessels; 2 = dilation of the iris and conjunctival vessels with moderate fare in the anterior chamber; 3 = intense iridal hyperemia associated with intense flare in the anterior chamber; and 4 = same clinical signs as 3 in addition to the presence of fibrinous exudates or syncehiae in the pupil.

### Scotopic Electroretinographic (ERG) Analysis

Twenty-four hours after LPS-administration, scotopic ERGs were used to evaluate the previously described retinal function (Lin et al., 2017). Recordings were obtained using 10-ms flash stimuli with an intensity of 19.1 cd.s/m^2^. A standard scotopic ERG consists of an a-wave and b-wave (Perlman, 1983). The amplitude of the ERG a-wave was measured from baseline to the negative peak, and the b-wave amplitude was measured from the trough of the a-wave to the peak of the positive wave. The temporal properties of the ERG response were identified by the time-to-peak (implicit time) for the a- and b-waves, which are measured from the stimulus onset.

### Determination of the Protein Concentration and Cytokine/Chemokine Levels in Aqueous Humor and Supernatant From Cultured BV-2 Microglial Cells

Aqueous humor was collected by anterior chamber puncture with a 30-gauge needle after the rat was euthanized 24 h after LPS injection. The total protein concentration in the aqueous humor was measured by Bio-Rad Protein Assay (Bio-Rad Laboratories, Hercules, CA, United States). The PGE_2_, TNF-α and MCP-1 levels of the aqueous humor or supernatant from cultured BV-2 microglial cells were examined by enzyme-linked immunosorbent assay (ELISA) with commercial kits (Cayman, Ann Arbor, MI, United States; BioLegend, San Diego, CA, United States and eBioscience, Vienna, Austria, respectively).

### Gelatin Zymography Analysis

MMP-9 expression and activation in aqueous humor were evaluated by gelatin zymography as previously described (Chou et al., 2010). Clear zones (bands) in a blue background gel represented the MMP-9 degradatory activity.

### Western Blot Analysis

Samples of whole eye ball homogenates and microglia cell lysates were collected and lysed as described (Lin et al., 2017), followed by SDS-PAGE separation and transfer onto nitrocellulose membranes. The membranes were blocked with 5% non-fat milk in TBS with 0.1% Tween 20 for 30 min and incubated with indicated primary antibodies at 4°C overnight. The membranes were then washed and incubated with HRP-conjugated secondary antibodies for 1 h, which was followed by detection of the immunoreactive bands with ECL. The ratio of the optical density of the protein product to the internal control (β-actin) was obtained and expressed as a ratio.

### Immunofluorescent Evaluation

The eyes sections were obtained from the euthanized rats at 24 h after LPS-injection and processed as previously described (Lin et al., 2017). The sections were incubated with anti-GFAP (1:100) or anti-Iba1 (1:50) at 4°C overnight. Then, the sections were rinsed and subsequently incubated with the fluorescence-conjugated secondary antibody Dylight 488 (1:200) for 2 h. After washes, the sections were mounted on cover slips using HIGHDEF^®^ IHC fluoromount contained 2 μM DAPI. The retinal sections were detected with a Leica TCS SP5 confocal microscope imaging system (Leica Microsystems, Wetzlar, Germany).

### Protein Oxidation Measurement

Protein oxidation was measured in eye homogenates. The carbonyl group derived from oxidative proteins is considered a biomarker of protein oxidation status (Levine et al., 1994). The Oxyblot protein oxidation detection kit (Millipore, Billerica, MA, United States) was used to detect the protein carbonyl groups. Briefly, 20 μg of total protein was denatured with 6% SDS (final concentration), which was followed by derivatization of exposed carbonyl groups with 2,4-dinitrophenylhydrazine (DNPH) to form 2,4-dinitrophenylhydrazone (DNP-hydrazones) by adding 10 μl of 1 × DNPH solution. After incubation at room temperature for 15 min, 7.5 μl of the Neutralization solution was added to stop the reaction. The DNP-derivatized samples were subjected to Western blot analysis using primary antibody specific to the DNP-moiety of the oxidatively modified proteins.

### Cell Culture

The mouse BV-2 microglia cell line was cultured in DMEM supplemented with penicillin (90 units/ml), streptomycin (90 μg/mL), l-glutamine (3.65 mM), HEPES (18 mM), NaHCO_3_ (23.57 mM), and 10% heat-inactivated fetal bovine serum (FBS) at 37°C in a humidified atmosphere (95% O_2_ and 5% CO_2_). The cell culture conditions and treatments have been previously described (Wang et al., 2014).

### MTT Assay

Cell viability was measured using colorimetric MTT assay. In brief, BV-2 cells were seeded in 12-well plate at a density of 1 × 10^6^ and incubated with various concentrations of LC53 (2, 5, 10 μM) for 22.5 h. After treatment, MTT (0.55 mg/ml) was added and further incubated for 1.5 h. Then the cells were lysed in 1 ml DMSO. The absorbance values at 550 nm were measured on a microplate reader (Thermo Multiskan GO, Ratastie, Finland).

### Measurement of Nitric Oxide (NO) Production

Nitric oxide level in the culture supernatant was measured using nitrate/nitrite colorimetric assay kit (Cayman, Ann Arbor, MI, United States). BV-2 cells were seeded at a density of 1 × 10^6^ in 12-well plate. After LPS-stimulation for 24 h in the presence or absence of LC53, the culture supernatants were collected to measure NO production. 100 μl culture supernatant was mixed with 50 μl Griess regent A and 50 μl Griess regent B sequentially and incubated for 20 min. Absorbance values at 550 nm were measured on a microplate reader (Thermo Multiskan GO, Ratastie, Finland) and nitrite concentrations were calculated by comparison to the nitrite standard.

### Measurement of ROS Production

Intracellular ROS production of BV-2 microglia was evaluated by detecting the fluorescent intensity of 2′, 7′-dichlorofluorescein (DCF), the oxidized product of the 2′, 7′-dichlorodihydrofluorescein diacetate (DCFH-DA), by flow cytometry. The fluorescence intensity represents the level of ROS production in the cells. Briefly, BV-2 cells were seeded in 12 wells plate at the density of 1.0 × 10^6^ with FBS (0.5%)-contained DMEM medium. After 17 h required for quiescence, cells were incubated with LC53 (10 μM) for 30 min followed by LPS (150 ng/ml) stimulation for 24 h. Subsequently, the cells were trypsinized and collected by centrifugation at 1000 RPM for 5 min. The dissociated cells were re-suspended in 0.5 ml of DMEM (without phenol red) containing 10 μM DCFH-DA and allowed to react for 40 min. After incubation, the DCF fluorescence intensities of 10,000 cells were analyzed by a flow cytometer (BD Accuri^TM^ C6, Franklin Lakes, NJ, United States) with excitation at 488 nm and emission at 533/30 nm, and the data were acquired with BD Accuri^TM^ C6 software. The ROS production level was considered directly proportional to the fluorescence intensity.

### Preparation of Cytosolic and Nuclear Fractions

Nuclear and cytoplasmic extracts were prepared with Nuclear extract kit (Signosis, Santa Clara, CA, United States). BV2 microglial cells (1 × 10^6^ cells/m1) were seeded in 10 cm dish. Cells were pretreated with LC53 (10 μM) or parthenolide (10 μM) for 15 min prior to the treatment of LPS (150 ng/ml). After the co-incubation for 30 min, cells were scraped with 1 ml cold PBS and each fraction was lysed according to the manufacturer’s instructions.

### Reverse Transcription-Polymerase Chain Reaction (RT-PCR)

Total RNA was isolated from BV-2 using the TRIsure^TM^ reagent and RNA (0.5 μg) was reverse-transcribed to produce cDNAs and amplified using One-tube RT-PCR mix. RT-PCR was conducted using the following primers: mouse iNOS (310 bp) sense: 5′-CTG CAG CAC TTG GAT CAG GAA CCT G-3′, antisense: 5′-GGG AGT AGC CTG TGT GCA CCT GGA A-3′; and mouse β-actin (287 bp) sense: 5′-ATC CTG AAA GAC CTC TAT GC-3′, antisense: 5′-AAC GCA GCT CAG TAA CAG TC-3′. The PCR was performed with the following conditions: 25 cycles of a 30-s denaturation step at 94°C, a 30-s annealing step at 60°C, and a 20-s extension step at 72°C to amplify iNOS cDNA, followed by 30 cycles of a 30-s denaturation step at 94°C, a 30-s annealing step at 46°C, and a 20-s extension step at 72°C to amplify β-actin cDNA. Following amplification, the PCR products were electrophoresed on 1.5% agarose gel and visualized by GelRed^TM^ staining. β-actin was used as internal control.

### Statistical Analysis

The experimental results are expressed as the mean ± SEM from the indicated number of experiments. The results were analyzed using one-way analysis of variance (ANOVA) with the Sigma Stat v3.5 software. The Student–Newman–Keuls test was used to evaluate statistically significant differences between the groups. A *p*-value < 0.05 was considered to be statistically significant.

## Results

### LC53 Inhibited Anterior Ocular Inflammation and Prevented the Retinal Function Deficiency of Electroretinography (ERG) in Experimental EIU

First, we examined whether LC53 could protect against ocular inflammation during EIU. Ophthalmic examination showed the development of hyperemia and edema associated with miosis in the iris 72 h after LPS (1 mg/kg) injection (**Figure 1B**). These clinical features were not observed in the controls, and they appeared less severe in the LC53 (20 mg/kg) and LPS-treated rats. Quantitative evaluation of these clinical scores (**Figure 1C**) showed a significant increase in animals injected with LPS (3.50 ± 0.29) compared with the control group (0.25 ± 0.25), and the LPS-injured animals had a significant reduction in the clinical scores in the presence of LC53 (0.75 ± 0.48).

To assess whether LC53 treatment could restore photoreceptor cell function, we measured the ERG scotopic responses in EIU rats. After 24 h of the induction of EIU, the a-wave and b-wave amplitudes were significantly reduced in EIU retinas (LPS 1 mg/kg; a-wave, 107.7 ± 19.9 μV; b-wave, 253.9 ± 28.5 μV) compared with the control group (a-wave, 222.6 ± 18.5 μV; b-wave, 589.4 ± 32.3 μV). LC53 (20 mg/kg) treatment rescued LPS-induced retinal dysfunction and increased the ERG responses (a-wave, 145.2.0 ± 12.5 μV and b-wave, 396.9 ± 42.5 μV) (**Figures 1D,E**). Moreover, a delayed implicit time was found in the b-wave of the LPS-injured group [96.0 ± 5.9 milliseconds (ms)] compared with the control group (84.1 ± 2.8 ms) (**Figures 1D,F**). LC53 treatment improved the ERG responses (implicit time of b-wave: 84.6 ± 1.8 ms) and protected against LPS-induced visual impairment.

### LC53 Attenuated LPS-Induced Cytokine Production, Protein Leakage, and Matrix Metalloproteinase (MMP) Activation of EIU Rats

**FIGURE 2:**
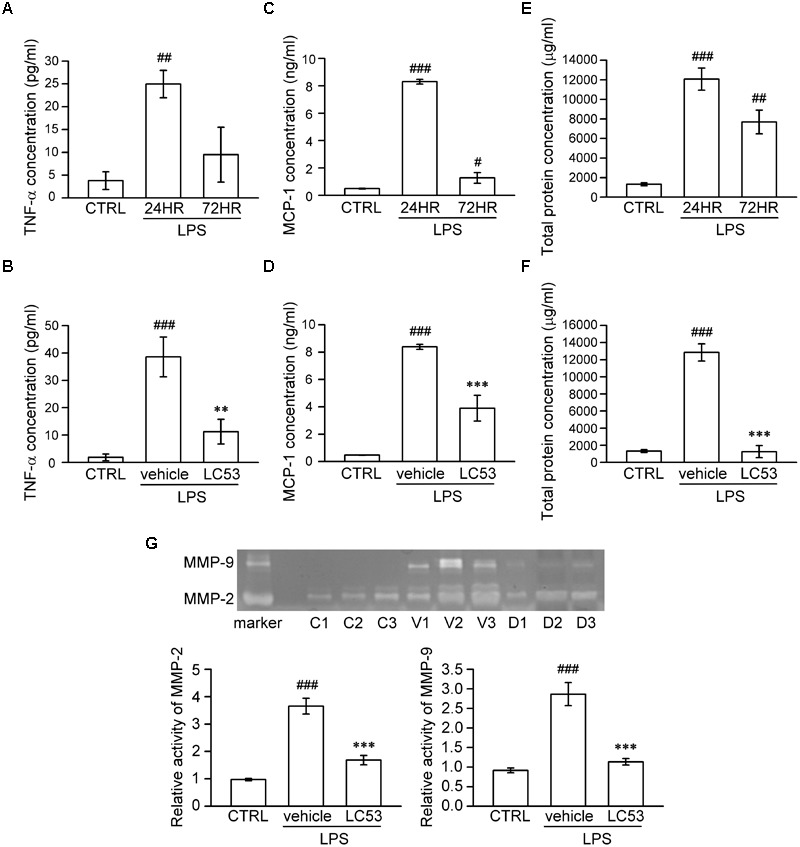
LC53 decreased the levels of TNF-α and MCP-1, protein exudation, and activation of MMP-2 and MMP-9 in rat aqueous humor. Fresh aqueous humor was obtained by anterior chamber puncture with a 30-gauge needle from euthanized rat. **(A,C,E)** Quantification of the time courses of MCP-1, TNF-α, and protein amount of aqueous humor, indicating that anterior ocular inflammation peaked at 24 h after LPS (1 mg/kg)-injection in aqueous humor. **(B,D,F)** Quantification of the average amount of TNF-α, MCP-1, and protein amount from control and EIU rat 24 h after LPS-injection in the presence or absence of LC53 (20 mg/kg). **(G)** MMP-9 gelatinolytic activity in aqueous humor was evaluated by zymography. The referenced markers were from medium of HT-1080 cell line, which constitutively secreted MMP-2 and MMP-9. The quantification values were presented as the mean ± SEM of results from 4 to 6 animals. Representative bands from 3 independent experiments were shown for each group: the control groups (C1, C2, C3), vehicle-treated EIU groups (V1, V2, V3), and LC53-treated EIU groups (D1, D2, D3). CTRL: control. ^#^*p* < 0.05, ^##^*p* < 0.01, ^###^*p* < 0.001 compared with the control group treated with normal saline; ^∗∗^*p* < 0.01, ^∗∗∗^*p* < 0.001 compared with the EIU group treated with vehicle.

It is suggested that analysis of aqueous humor, such as the chemokine, cytokine, MMP levels, and total leucocyte numbers, may be a reliable gauge for estimating inflammation (Cuello et al., 2002). Accordingly, we investigated the anti-inflammatory effect of LC53 on the anterior section of the eye, examining the proinflammatory mediators (TNF-α and MCP-1), protein concentration, and activation of MMPs in aqueous humor. First, we examined different induction times after LPS-injection and found the levels of TNF-α, MCP-1, and protein concentration peaked at 24 h after LPS-injection (24.96 ± 2.99 pg/ml; 8.30 ± 0.17 ng/ml; and 12067.83 ± 1130.79 μg/ml) compared with the controls (3.78 ± 1.94 pg/ml; 0.49 ± 0.03 ng/ml; and 1325.49 ± 156.05 μg/ml) and the 72 h LPS-inflamed groups (9.48 ± 6.02 pg/ml; 1.28 ± 0.39 ng/ml; and 7693.68 ± 1215.79 μg/ml) (**Figures 2A,C,E**), respectively. The dramatically increased protein concentration represented protein leakage or exudation. Treatment with LC53 (20 mg/kg) in LPS-injured animals significantly reduced the level of TNF-α and MCP-1, and protein concentration (11.24 ± 4.52 pg/ml; 3.90 ± 0.94 ng/ml, and 1253.17 ± 705.88 μg/ml) compared with the EIU groups (38.59 ± 7.26 pg/ml; 8.38 ± 0.18 ng/ml; and 12846.86 ± 1003.83 μg/ml) (**Figures 2B,D,F**). Elevated levels of MMPs were found in the aqueous humor of patients with uveal inflammation and of animals with LPS-inflammation. These findings indicated that gelatinases and other MMPs served as the pathogenic factor of uveitis (Di Girolamo et al., 1996). Herein, we evaluated the MMP-2 and MMP-9 activity in the aqueous humor using gelatin zymography. At 24 h after LPS-injection, the activities of MMP-2 and MMP-9 were dramatically increased (3.65 ± 0.29 and 2.86 ± 0.29) compared with control groups (0.97 ± 0.04 and 0.92 ± 0.06). MMP-2 and MMP-9 activation was significantly suppressed (1.68 ± 0.17 and 1.14 ± 0.08) in the presence of LC53 in EIU rats (**Figure 2G**).

### LC53 Ameliorated the Extension of Glial Fibrillary Acidic Protein (GFAP) and Inhibited the Accumulation of Iba-1 in EIU Rats

**FIGURE 3:**
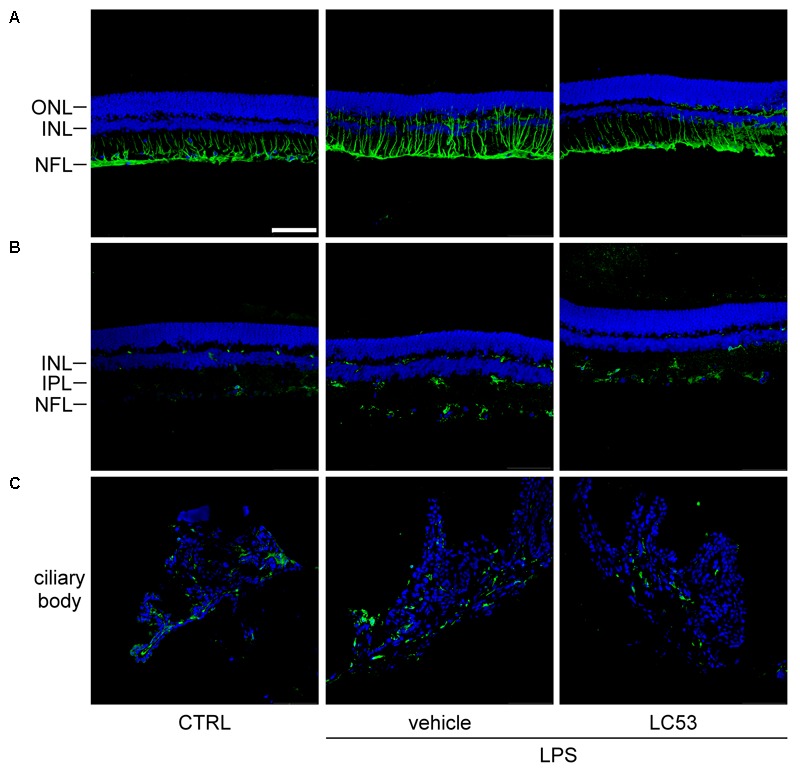
LC53 suppressed the reactivity of Müller glial cells and attenuated the accumulation of monocyte/macrophage in ciliary body and retina section. Representative immunofluorescent images were acquired by confocal from cryo-sectioned eye tissue slices. **(A)** Activity of Müller glial cells were visualized by GFAP-labeling signal (green) in retinal sections counterstained with DAPI (blue). Distribution of monocyte/macrophage was visualized by Iba-1-labeling signal (green) in retinal sections **(B)** and ciliary body **(C)** counterstained with DAPI (blue). CTRL, control; ONL, outer nuclear layer; INL, inner nuclear layer; NFL, nerve fiber layer; IPL, inner plexiform layer. Scale bar: 75 μm.

GFAP expression has long been recognized as a marker of pathologic change in Müller glial cells (Eisenfeld et al., 1984). In the resting phase, GFAP localization is primarily restricted to the nerve fiber layer and ganglion cell layer. Immunofluorescence assays demonstrated that GFAP-positive processes were prolonged and extended from nerve fiber layer to inner nuclear layer, and GFAP protein levels were obviously increased in EIU rats (LPS, 1 mg/kg) (**Figure 3A**). It was postulated that reactive Müller glial cells may dysregulate the microenvironment of the retina. This effect was apparently inhibited by LC53 treatment (**Figure 3A**) in LPS-injured rats. Iba-1 expression is mostly limited to the macrophage/monocyte lineage (Nakamura et al., 2013). Immunostaining for Iba-1 showed increased intensity along level changes in macrophage/monocyte populations in the retina (posterior section) (**Figure 3B**) and ciliary body (anterior section) (**Figure 3C**) during EIU. Moreover, more macrophage/monocyte populations were found to migrate toward the INL of retina. EIU rats treated with LC53 showed the suppression of Iba-1 accumulation in the retina and ciliary body. All slides were DAPI-counterstained to identify the retinal layers and anterior ciliary-body.

### LC53 Suppressed p65 Phosphorylation, HSP90 Expression, and Oxidative Stress as Well as Exerted the Anti-apoptotic Activity in EIU Rats

**FIGURE 4:**
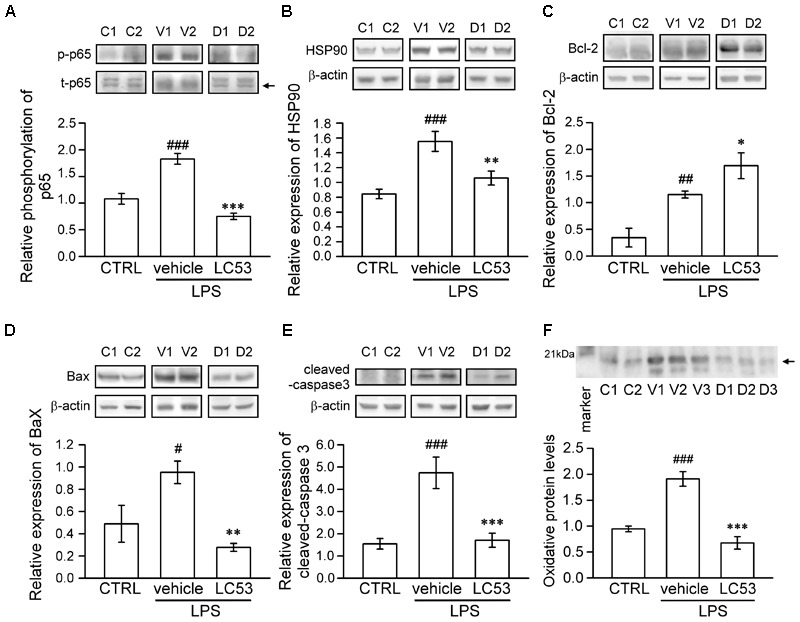
LC53 exerted anti-inflammatory and anti-apoptosis properties during EIU. Phosphorylation of p65 **(A)**, HSP90 **(B)**, Bcl-2 **(C)**, Bax **(D)**, and cleaved-caspase-3 **(E)** in eye homogenates were analyzed using Western blot. **(F)** Eye homogenates were examined for the protein oxidation using Oxyblot. Primary antibody specific to DNP-moiety was used to target the oxidatively modified proteins in Western blot. The specific marker (21 kDa) was shown on the left side, which represented carbonylated-trypsin inhibitor from the mixture of standard proteins. The quantification values were presented as the mean ± SEM of results from 5 to 7 animals. Representative bands from 2 to 3 independent experiments were shown for each group: the control groups (C1, C2), vehicle-treated EIU groups (V1, V2, V3), and LC53-treated EIU groups (D1, D2, D3). C, CTRL: control; V: vehicle.^#^*p* < 0.05, ^##^*p* < 0.01, ^###^*p* < 0.001 compared with the control groups treated with normal saline; ^∗^*p* < 0.05, ^∗∗^*p* < 0.01, ^∗∗∗^*p* < 0.001 compared with the EIU group treated with vehicle.

To identify the mechanisms underlying the ocular-protective effect of LC53, we examined the phosphorylation of p65 and expression of HSP90, Bcl-2, Bax, and cleaved-caspase-3 in the eye homogenates of EIU (**Figure 4**). The phosphorylation level of p65 was 1.7-fold higher in EIU rats compared with normal controls (1.83 ± 0.10 versus 1.08 ± 0.10), while the presence of LC53 in EIU rats substantially decreased the p65 phosphorylation level of 0.79 ± 0.75 (**Figure 4A**). In addition, HSP90 inhibition supported the anti-inflammatory effects through suppressing leukocyte adhesion and blood-retinal barrier breakdown in EIU models (Poulaki et al., 2007). Also, HSP90 is required for the folding and stability of TAK1 (Liu et al., 2008), which functions upstream of both IKK and MAPK. Therefore, we further examined the expression of HSP90. The HSP90 levels increased 1.8-fold in EIU rats compared with normal controls (1.55 ± 0.13 versus 0.84 ± 0.06). Additionally, the HSP90 levels significantly decreased in the LC53-treated EIU rats (1.06 ± 0.09) (**Figure 4B**). Although the LPS-induced inflammation in EIU resolves spontaneously after several days, it induces apoptosis within the eye (Yang et al., 2003). Consistent with this finding, we revealed that the Bax and cleaved-caspase-3 levels were increased 1.9- and 3.1-fold in EIU rats (0.95 ± 0.10 and 4.74 ± 0.71) compared with control retinas (0.49 ± 0.17 and 1.55 ± 0.24), and LC53 significantly reduced the Bax and cleaved-caspase-3 levels in EIU rats by 3.4- and 2.8-fold (0.28 ± 0.04 and 1.71 ± 0.31) compared with the EIU groups, respectively (**Figures 4D,E**). In addition, Bcl-2 levels of eye homogenates was increased in EIU animals compared with normal controls (1.15 ± 0.06 versus 0.35 ± 0.17). LC53 treatment could enhance the Bcl-2 levels of 1.5-fold (1.69 ± 0.24) in EIU rats compared with the vehicle-treated EIU group (**Figure 4C**). Together, these results revealed that LC53 served as an anti-inflammatory agent through inactivating NF-κB and suppressing HSP90. Moreover, the anti-apoptotic effect of LC53 was mediated by the down-regulation of Bax and caspase-3 and induction of Bcl-2 in the EIU model.

Furthermore, oxidative stress likely plays a causative role in both non-infectious and infectious uveitis (Yadav et al., 2011). To identify the variety of oxidative stress in the eye, we examined the protein oxidation of the eye homogenates, detected as carbonylated proteins, using an Oxyblot kit. LPS-injection apparently increased the oxidatively modified proteins (1.91 ± 0.14), representing oxidative stress compared with normal control group (0.95 ± 0.05). LC53 significantly decreased the reactive oxidant burden (0.68 ± 0.12) after LPS-injection (**Figure 4F**).

### LC53 Inhibited the Inflammatory Enzymes and Mediators in LPS-Stimulated Microglial BV2 Cells

**FIGURE 5:**
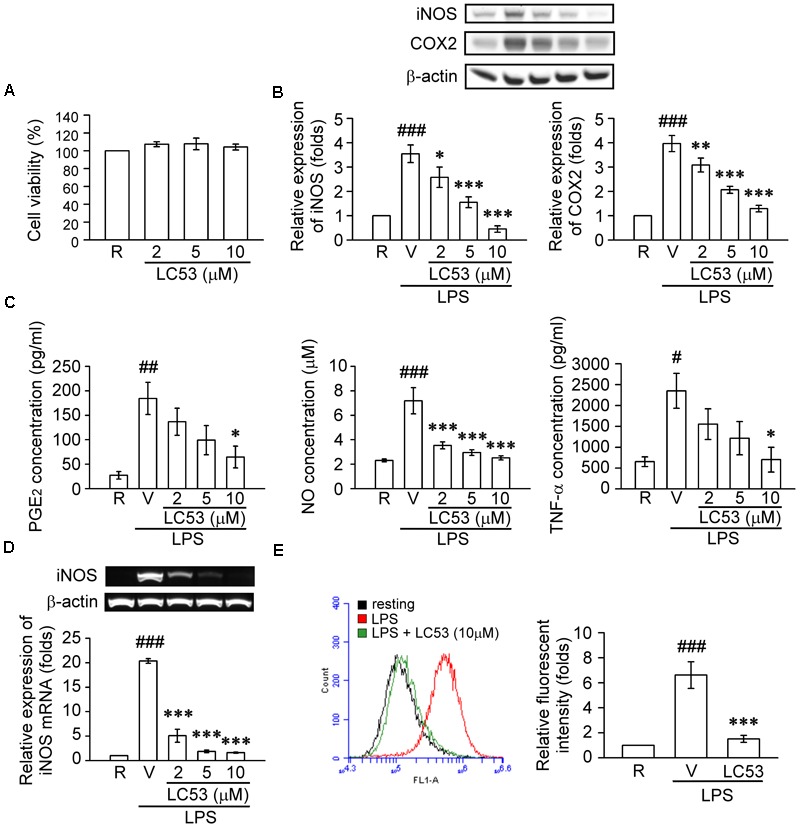
LC53 inhibited the productions of inflammatory enzymes and mediators in LPS-stimulated microglial BV2 cells. BV2 cells were pretreated with the vehicle (DMSO) or LC53 (2, 5, and/or 10 μM) for 30 min and stimulated with LPS (150 ng/ml) for indicated time. **(A)** Colorimetric MTT assay was used to measure BV-2 cell viability after LC53 incubation for 24 h. The percentage of the absorbance was compared to the resting group treated with DMSO. **(B)** Protein level of iNOS and COX-2 in cell lysates were examined 24 h after LPS-stimulation using Western blot. **(C)** The PGE_2_, NO and TNF-α level of supernatants from cultured BV-2 microglial cells were evaluated 24 h after LPS-stimulation by ELISA or nitrate/nitrite colorimetric assay kit. **(D)** Expression of iNOS mRNA was evaluated 6 h after LPS stimulation by RT-PCR analysis. β-actin was used as internal control. **(E)** After LPS-stimulation for 24 h, BV-2 microglial cells were incubated with DCFH-DA for 40 min. Then the DCF fluorescent intensities were analyzed by flow cytometry. The results were analyzed from 3 to 6 independent experiments and presented as the mean ± SEM. R, resting; V, vehicle (DMSO). ^#^*p* < 0.05, ^##^*p* < 0.01, ^###^*p* < 0.001 compared with the resting group; ^∗^*p* < 0.05, ^∗∗^*p* < 0.01, ^∗∗∗^*p* < 0.001 compared with the LPS-stimulated group treated with vehicle.

Previous findings suggested that retinal microglia initiates retinitis with subsequent recruitment of circulatory inflammatory cells during EAU (Rao et al., 2003). Chen et al. (2009) also revealed that TLR-4 was mainly expressed in the surface of iris-ciliary macrophages during EIU. Therefore, a mouse microglial BV-2 cell line was stimulated with LPS, a TLR-4 agonist, to validate the anti-inflammatory effects and mechanisms of LC53. Accordingly, we evaluated the anti-inflammatory effects through iNOS, COX-2, NO and PGE_2_ levels, which are considered major markers of the activation of macrophages or microglia during inflammation (Yoon et al., 2018). In this cellular study, we demonstrated that BV2 cellular viability was not affected by LC53 at various concentrations (**Figure 5A**). LPS (150 ng/ml) significantly increased the iNOS and COX-2 protein levels in BV2 microglial cells, and LC53 (2, 5, and 10 μM) concentration-dependently inhibited LPS-induced iNOS and COX-2 expression (**Figure 5B**). Moreover, LC53 significantly suppressed the PGE_2_, NO and TNF-α production from LPS-stimulated BV2 microglial cells (**Figure 5C**). The effects of LC53 on iNOS mRNA expression were evaluated to clarify whether LC53 suppressed the LPS-mediated induction of iNOS at the pre-translational level. RT-PCR data revealed that the reduction in iNOS mRNA correlated with the reduction in the iNOS protein level (**Figure 5D**). In addition, the effect of LC53 on ROS production in LPS-treated BV-2 cells was examined using the DCFH-DA fluorescent probe. The DCF fluorescence intensities representing the intracellular ROS levels were analyzed by flow cytometry. LPS stimulation triggered an increase in the cellular ROS levels (6.6 ± 1.1, expressed as relative folds) compared with the resting levels (1.0 ± 0.0). This effect was significantly reduced by LC53 treatment at a concentration of 10 μM (1.4 ± 0.2) (**Figure 5E**).

### LC53 Down-Regulated NF-κB and MAPKs Signaling in LPS-Stimulated Microglial BV-2 Cells

**FIGURE 6:**
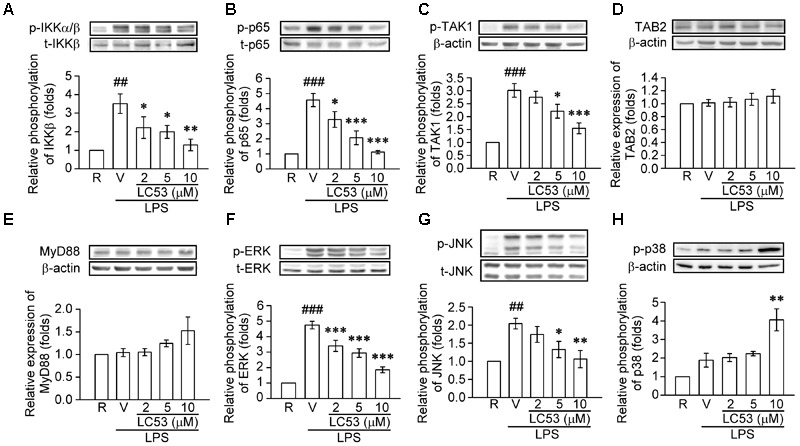
LC53 abrogated the NFκB and MAPKs signaling in LPS-stimulated microglial BV2 cells. Microglial BV2 cells were pretreated with the vehicle (DMSO) or LC53 (2, 5, and 10 μM) and stimulated with LPS (150 ng/ml) for indicated time. Phosphorylated level of IKKα***/***β (**A**, *n* = 4), p65 (**B**, *n* = 4), TAK1 (**C**, *n* = 6), ERK (**F**, *n* = 7), JNK (**G**, *n* = 5), and p38 (**H**, *n* = 4), and protein level of TAB2 (**D**, *n* = 7) and MyD88 (**E**, *n* = 3) in cell lysates were examined using Western blot. R, resting; V, vehicle (DMSO). ^##^*p* < 0.01, ^###^*p* < 0.001 compared with the resting group; ^∗^*p* < 0.05, ^∗∗^*p* < 0.01, ^∗∗∗^*p* < 0.001 compared with the LPS-stimulated group treated with vehicle.

**FIGURE 7:**
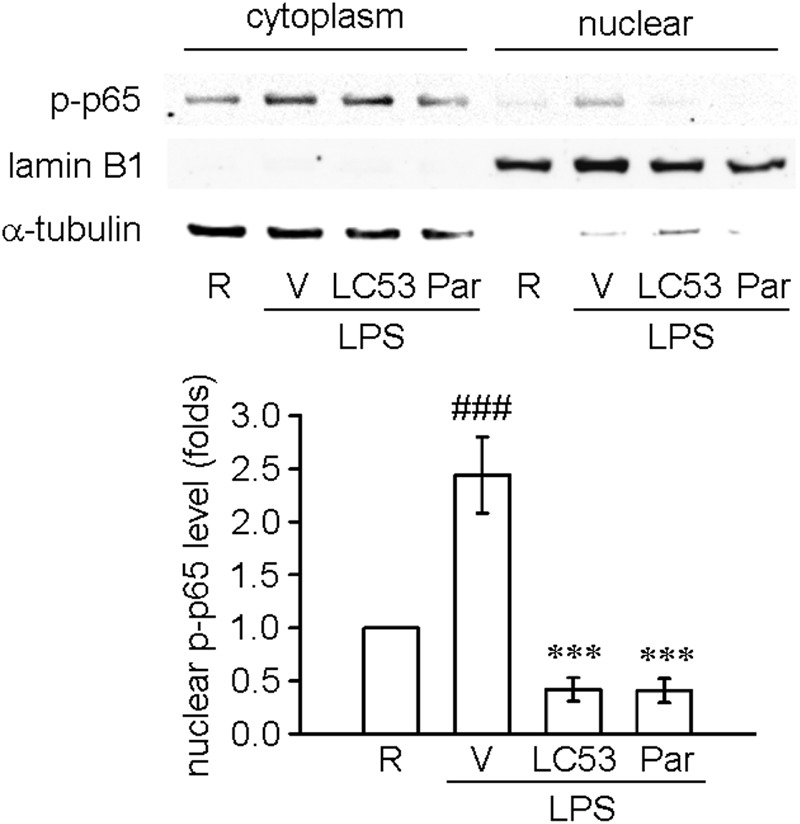
LC53 inhibited the nuclear phosphorylated p65 translocation in LPS-stimulated microglial BV2 cells. Microglial BV2 cells were pretreated with the vehicle (DMSO), LC53 (10 μM) or parthenolide (10 μM) and stimulated with LPS (150 ng/ml) for 30 min. Phosphorylated p65 levels in both cytoplasm and nuclear of BV2 microglia were examined using Western blot. The detections of α-tubulin and lamin B1 were used as the internal control for cytoplasm and nucleus (*n* = 4). R, resting; V, vehicle (DMSO); Par, parthenolide; p-p65, phosphorylated p65. ^###^*p* < 0.001 compared with the resting group; ^∗∗∗^*p* < 0.001 compared with the LPS-stimulated group treated with vehicle.

TLR-4 recognizes LPS and initiates a signaling cascade through the Toll/IL-1R (TIR) domain, cooperating with MyD88, which activates IL-1R-associated kinases (IRAKs). The IRAKs-contained complex further interacts with a preformed complex consisting of TAK1 and its adaptors TAB1 and TAB2. Activated TAK1 leads to downstream activation of IκB kinases (IKKs) and mitogen activated protein kinases (MAPKs) (Schauvliege et al., 2006). First, we examined the ability of LC53 to inhibit the activation of NF-κB in LPS-induced BV2 microglial cells. Exposure to LPS increased IKKβ and p65 phosphorylation, and these effects were suppressed by LC53 in a concentration-dependent manner (**Figures 6A,B**). We also investigated effect of LC53 on phosphorylation of TAK1 in LPS-stimulated BV2 microglial cells. Stimulation with LPS significantly induced phosphorylation of TAK1, and LC53 treatment markedly and strongly inhibited LPS-induced TAK1 phosphorylation (**Figure 6C**) without affecting the expression levels of TAB2 and MyD88 (**Figures 6D,E**). On the other hand, ERK and JNK phosphorylation were concentration dependently suppressed (**Figures 6F,G**) by LC53 with LPS stimulation under experimental condition. By contrast, LC53 enhanced the phosphorylation of p38 MAPK at a high concentration of 10 μM compared with vehicle group (**Figure 6H**). In addition, phosphorylated p65 protein has to be translocated from the cytoplasm to nucleus to initiate the transcription activity of downstream pro-inflammatory genes (Luo et al., 2018). Thus, we examined the levels of phosphorylated p65 in nuclear fractions of BV2 microglial cells. LPS (150 ng/ml) significantly increased the phosphorylated p65 levels in nuclear fractions (2.4 ± 0.4, expressed as relative folds) compared with the resting levels (1.0 ± 0.0). LC53 (10 μM) dramatically inhibited nuclear phosphorylated p65 translocation (0.4 ± 0.1), which was similar to the classical NF-κB inhibitor parthenolide (10 μM) (0.4 ± 0.1) (**Figure 7**).

## Discussion

Uveitis is a leading cause of legal blindness, which may arise from infections and autoimmune diseases such as Behçet’s disease and sarcoidosis. The major therapeutic strategies for uveitis involve controlling the inflammation and maintaining the visual function of the eye (Durrani et al., 2004; Yanai et al., 2014). The dysregulated innate immunity caused impairment in visual function in EIU detected by ERG (Kamoshita et al., 2016). Increased apoptosis levels were observed in the inner nuclear layers, which mainly consisted of Müller cells and ON bipolar cells, contributing to b-wave deficiency (Perlman, 1983; Koizumi et al., 2003). In addition, activation of NF-κB reduced the level of rhodopsin, an important visual substance in the photoreceptors, leading to visual function impairment with a-wave dysfunction (Perlman, 1983; Kamoshita et al., 2014). Similarly, our results revealed that the functional deficiencies of the a- and b-waves of ERG occurred 24 h after LPS injection in SD rats. LPS administration decreased the amplitude of both the a- and b-waves, and it mildly prolonged the implicit time of the b-wave. LC53 treatment significantly prevented the function deficiency of the b-wave representing the protection of the inner layer cells, and these effects may also occur through its anti-apoptosis property, including the inhibition of cleaved-caspase-3 and Bax expression, as well as the induction of anti-apoptotic protein Bcl-2.

During uveitis, Müller glial cells alter their characteristics to become reactive glial cells, leading to breakdown of the blood-retinal barrier with leukocyte infiltration into the vitreous cavity and leukocyte adhesion to the retinal vessels (Bhattacherjee et al., 1983), and it can be recognized by GFAP upregulation (Eisenfeld et al., 1984). Subsequent penetration of chemotactic leukocytes through basement membranes has been found depending on proteolysis of the extracellular matrix by MMP-2 and MMP-9 (El-Shabrawi et al., 2004). In the clinical setting, only MMP-2 was found in the control group, while higher levels of MMP-2 and -9 were detected in patients with higher uveitis activity (El-Shabrawi et al., 2000). Furthermore, MMP-2, as an activating sheddase of TNF-α, facilitates the secretion of active TNF-α, contributing to the chronicity of uveitis (El-Shabrawi et al., 2000; De Groef et al., 2015). Our results revealed that LC53 significantly inhibited LPS-induced reactivity of Müller glial cells (visualized by increased GFAP expression) and activation of MMP-9 and MMP-2, which may prevent blood-retinal barrier breakdown and previously described penetration of monocyte/macrophage populations.

TLR-4 is expressed in monocytes/macrophages, retinal pigment epithelial cells, and ocular endothelial cells (Shen et al., 2014). Increased expression of TLR4 and activation of NF-κB were found in the EIU rats (Li et al., 2010), and down-regulation of NF-κB signaling protected against endotoxin-induced injuries (Shen et al., 2014). Consistently, we found that LC53 down-regulated NF-κB activation in EIU rat eyes. On the other hand, Chen et al. (2009) observed that macrophages within the iris and ciliary body were the major populations expressing TLR-4 in EIU, and they were reported to be the major effectors of tissue damage in uveitis. Macrophages, as effectors of innate immunity, are active phagocytes that can release ROS, reactive nitrogen radicals, and antibacterial enzymes. In addition, macrophages serve as inductors of acquired immunity by secreting adhesion molecules and proinflammatory mediators (cytokines and chemokines) (Gordon, 2007). Accordingly, the progression of retinal pathologies is also ascribed to the reactivity of macrophages (Fernando et al., 2016). In the present study, LC53 treatment suppressed the infiltration of Iba-1 positive macrophages/monocytes in the anterior ciliary body and posterior retina segment during EIU. It is worth noting that resident microglia migrated toward the photoreceptor cell layer before circulating macrophages and polymorphonuclear cells in experimental uveitis, which could generate numerous neurotoxic agents, such as TNF-α and NO. These findings suggested that retinal microglia may initiate the retinitis, allowing for subsequent recruitment of circulation-derived inflammatory cells during EAU (Rao et al., 2003). Herein, we examined the anti-inflammatory effects of LC53 in LPS-activated BV-2 microglial cell line. Our results revealed that LC53 significantly inhibited LPS-induced iNOS and COX-2 expression, thereby reducing the production of NO and PGE_2_. Consequently, LC53 inactivated the microglia and suppressed the infiltration of monocyte/macrophage populations, which were beneficial to attenuate the initiation and amplification of immune responses in uveitis.

The transcription factor NF-κB is critical regulator of immunity and inflammation (Hoesel and Schmid, 2013). The degradation of IκB by activating IKKs releases NFκB (p65), and subsequent translocation of p65 to the nucleus expresses its proinflammatory function (Zhang and Sun, 2015). According to signal studies, LC53 inhibited LPS-induced IKKβ and p65 phosphorylation, and the pharmacological manipulation on IKKβ and p65 has been found to play a neuroprotective role during intraocular inflammation (Lennikov et al., 2012; Kamoshita et al., 2014). We also demonstrated that LC53 significantly inhibited nuclear phosphorylated p65 translocation in LPS-stimulated BV2 microglial cells. Furthermore, LC53 treatment strongly inhibited LPS-induced TAK1 phosphorylation, which is a key signaling component of NFκB and MAPK pathways (Ajibade et al., 2013). The inhibition of TAK1 has also been found to reduce inflammation and apoptosis in neurodegenerative diseases (Ridder and Schwaninger, 2013). Under LPS stimulation, the association of TAK1 and TAB2 was found to induce IKK phosphorylation, thereafter trigger NFκB activation (Imani Fooladi et al., 2011). We found the TAB2 content was not affected by LC53. It revealed that NFκB/TAK1 inhibition was not similar to TRIM38-mediated lysosomal-dependent TAB2/3 degradation (Hu et al., 2015). On the other hand, at higher concentration of LC53 (10 μM) could enhance p38 MAPK phosphorylation. It was proposed LC53 possibly regulated dual specific phosphatase 1 expression (Korhonen et al., 2011) or TLR2 activation (Zhou et al., 2017). Taken together, the anti-inflammatory mechanisms of LC53 may occur through inhibiting of NF-κB/TAK1/IKK signaling.

In addition to major inflammatory cells, various resident ocular cells can produce proinflammatory cytokines, including lens epithelium, ciliary body epithelium, RPE and retinal Müller cells. As cytokines/chemokines support an essential role in the immune response in uveitis, hypotheses to attenuate inflammation by antagonizing them have been studied (Heiligenhaus et al., 2010). During uveitis, the use of anti-TNF-α drugs was found to diminish corticoid doses, reduce the number of relapses, and improve disease control in clinical (Benitez-del-Castillo et al., 2005). On the other hand, the MCP-1 mutant or its receptor antagonists have been generated and found to be efficacious, alleviating inflammatory diseases (Piccinini et al., 2010), including EIU (Tuaillon et al., 2002). In the present study, we found that LC53 significantly reduced the levels of both TNF-α and MCP-1 in the aqueous humor, which may relieve subsequent leukocyte recruitment and amplify immune responses in EIU.

Oxidative stress is also implicated in the pathogenesis of uveitis. It was found that there were ocular lipid peroxidation products during experimental uveitis (Satici et al., 2003). Consistently, our results revealed increased oxidative stress in a LPS-induced EIU model and LPS-stimulated microglial BV-2 cells. The OxyBlot data revealed that some carbonylated proteins, with a molecular mass of approximately 20 kDa, had a positive tendency in accordance with the LPS-stimulation in the presence or absence of LC53. Accordingly, we predicted these proteins as α-crystallins according to their similar molecular weight and higher susceptibility to carbonylation under oxidative stress (Jahngen-Hodge et al., 1994). In mammalian lenses, α-crystallins account for approximately 50% of the protein mass (Huang et al., 1995). In addition to their major roles as structural and refractive lens proteins, α-crystallins serve as low molecular weight chaperones, protecting against various ocular diseases, including cataracts, retinitis pigmentosa, and macular degeneration (Christopher et al., 2014). In response to various stresses, α-crystallins have been found to bind to pro-apoptotic molecules Bax, Bcl-X(s), and p53, blocking their translocation from cytoplasm to mitochondria. Therefore, they inhibited the release of cytochrome c from mitochondria, cleavage of caspase-3, and prevented cell apoptosis (Mao et al., 2004). Moreover, α-crystallins protect against oxidative damage by scavenging peroxide and superoxide radicals, regulating the levels of glutathione and maintaining the activities of antioxidant enzymes (Masilamoni et al., 2005). Consistently, Piri et al. (2016) revealed that inhibition of HSP90 and overexpression of α-crystallins support the survival of injured RGCs in optic neuropathies. However, oxidized α-crystallins were recognized and degraded rapidly by cytoplasmic proteolytic systems (Huang et al., 1995). Based on these findings, we hypothesized that LPS administration increased the oxidation of α-crystallins in the eye, which exacerbated the cell apoptosis and oxidative stress burden. LC53 treatment reduced the oxidative levels of α-crystallins and inhibited HSP90 expression, which allowed for preservation of their chaperone-like protective effects in EIU.

## Conclusion

Our findings demonstrated that LC53 improved the retina deficiency and suppressed the inflammatory mediators of EIU. The cellular findings of inhibitions on TNF-α product, oxidative stress, and NF-κB signaling in microglia were consistent to *in vivo* studies. It was suggested that LC53 may be a potent therapeutic agent for retinal protection against ocular inflammatory diseases through its anti-inflammatory and anti-apoptotic activities. Further investigations for its long-term effects and possible therapeutic profiles in systemic inflammatory diseases associated with ocular inflammation are warranted.

## Author Contributions

F-LL, J-DH, T-HL, and GH designed the research. F-LL and J-LY carried out the experiments. F-LL, Y-WC, H-MC, and J-DH analyzed the results and prepared the figures. F-LL, GC, and GH wrote the manuscript. All authors reviewed and approved the final manuscript.

## Conflict of Interest Statement

The authors declare that the research was conducted in the absence of any commercial or financial relationships that could be construed as a potential conflict of interest. The reviewer MP and handling Editor declared their shared affiliation. The reviewer J-HJ declared a shared affiliation, though no other collaboration, with one of the authors T-HL to the handling Editor.

## Abbreviations

COX-2: cyclooxygenase-2
EIU: endotoxin-induced uveitis
ERG: electroretinography
GFAP: glial fibrillary acidic protein
iNOS: inducible nitric oxide synthase
LC53: theissenolactone C
LPS: lipopolysaccharide
MAPK: mitogen-activated protein kinase
MCP-1: monocyte chemoattractant protein-1
MMP: matrix metalloproteinase
NF-κB: nuclear transcription-factor kappa B
NO: nitric oxide
PGE_2_: prostaglandin E_2_
ROS: reactive oxygen species
TAB: TAK1 binding protein
TAK1: TGF-β-activated kinase 1
TLR-4: toll-like receptor-4
TNF-α: tumor necrosis factor-α
